# Genome-Wide Gene-Environment Interaction Analysis Using Set-Based Association Tests

**DOI:** 10.3389/fgene.2018.00715

**Published:** 2019-01-14

**Authors:** Wan-Yu Lin, Ching-Chieh Huang, Yu-Li Liu, Shih-Jen Tsai, Po-Hsiu Kuo

**Affiliations:** ^1^Institute of Epidemiology and Preventive Medicine, College of Public Health, National Taiwan University, Taipei, Taiwan; ^2^Department of Public Health, College of Public Health, National Taiwan University, Taipei, Taiwan; ^3^Center for Neuropsychiatric Research, National Health Research Institutes, Zhunan, Taiwan; ^4^Department of Psychiatry, Taipei Veterans General Hospital, Taipei, Taiwan; ^5^Division of Psychiatry, National Yang-Ming University, Taipei, Taiwan

**Keywords:** diastolic blood pressure, systolic blood pressure, hypertension, gene-alcohol interaction, Taiwan Biobank, multiple testing correction

## Abstract

The identification of gene-environment interactions (G × E) may eventually guide health-related choices and medical interventions for complex diseases. More powerful methods must be developed to identify G × E. The “adaptive combination of Bayes factors method” (ADABF) has been proposed as a powerful genome-wide polygenic approach to detect G × E. In this work, we evaluate its performance when serving as a gene-based G × E test. We compare ADABF with six tests including the “Set-Based gene-EnviRonment InterAction test” (SBERIA), “gene-environment set association test” (GESAT), etc. With extensive simulations, SBERIA and ADABF are found to be more powerful than other G × E tests. However, SBERIA suffers from a power loss when 50% SNP main effects are in the same direction with the SNP × E interaction effects while 50% are in the opposite direction. We further applied these seven G × E methods to the Taiwan Biobank data to explore gene× alcohol interactions on blood pressure levels. The *ADAMTS7P1* gene at chromosome 15q25.2 was detected to interact with alcohol consumption on diastolic blood pressure (*p* = 9.5 × 10^−7^, according to the GESAT test). At this gene, the *P*-values provided by other six tests all reached the suggestive significance level (*p* < 5 × 10^−5^). Regarding the computation time required for a genome-wide G × E analysis, SBERIA is the fastest method, followed by ADABF. Considering the validity, power performance, robustness, and computation time, ADABF is recommended for genome-wide G × E analyses.

## Introduction

“Gene-environment interaction” (G × E) is defined as “a different effect of an environmental exposure on disease risk in subjects with different genotypes” or “a different effect of a genotype on disease risk in subjects with different environmental exposures” (Ottman, 1996). “Gene-treatment interactions” are specific examples of G × E in pharmacogenomics. Searching for genes that may modify drug responses will significantly improve drug delivery by identifying subjects that can benefit from therapy and those at an increased risk of harm (He and Allen, 2010; Chen et al., 2011; Ko et al., 2015). The identification of G × E and gene-treatment interactions may eventually guide health-related choices and medical interventions for complex diseases (Franks and Pare, 2016). Clearly, more powerful methods must be developed to detect G × E (Hunter, 2005; Zhang and Biswas, 2015).

Exploring G × E is important for disease prevention. However, compared with the success achieved in identifying genetic main effects, very few G × E findings have been replicated partially due to the lack of power (Jiao et al., 2013). Single-nucleotide polymorphism (SNP) analysis (one SNP at a time) is a commonly used approach. Nonetheless, this approach suffers from a power loss due to a harsh penalty of multiple testing. Even true positives may not stand out under the stringent genome-wide significance level (Lin and Lee, 2010), i.e., 5 × 10^−8^.

Several set-based (or gene-based) analysis methods have been developed to aggregate the G × E signals within a gene/region and alleviate the multiple-testing penalty (Jiao et al., 2013; Lin et al., 2013, 2016; Chen et al., 2014). Jiao et al. proposed a two-stage “Set-Based gene-EnviRonment InterAction test” for case-control studies, called “SBERIA” (Jiao et al., 2013). During the first stage, the SNPs are filtered according to their associations with E (this is Step 1 in Murcray et al., 2009). The sign and significance of the filtering statistics are then used to weight SNP × E in the second stage (Jiao et al., 2013).

Lin et al. proposed the “gene-environment set association test” (GESAT), which is a variance component (VC) test that estimates the SNP main effects using a ridge regression (Lin et al., 2013). These authors later developed an “interaction sequence kernel association test” (iSKAT) that is regarded as the optimal in the class of VC tests (Lin et al., 2016). While GESAT assumes no correlation among the SNP × E interaction effects, iSKAT searches for the optimal correlation coefficient among them. Therefore, GESAT is a specific case of iSKAT, and both approaches can be implemented using the “iSKAT” R package.

Chen et al. proposed a G × E test that treats the SNP main effects as fixed (designated “INT_FIX”) or random (designated “INT_RAN”). They also developed a joint test for detecting the genetic associations while allowing for G × E (designated “JOINT”) (Chen et al., 2014). These three methods belong to the class of VC tests and can be performed using the “rareGE” R package.

The abovementioned methods have been proposed with user-friendly analysis tools that are popular choices for G × E analyses. Recently, the “adaptive combination of Bayes factors method” (ADABF) has been proposed as a powerful polygenic approach to detect G × E (Lin et al., 2018). This method can also serve as a gene-based G × E test. In this study, we evaluate the performance of ADABF when detecting gene-based G × E signals. We compare ADABF with the abovementioned six tests. Using a sample of 16,555 subjects from the Taiwan Biobank (TWB) data, we perform a genome-wide gene-alcohol interaction analysis on diastolic blood pressure (DBP) and systolic blood pressure (SBP). The validity, power, robustness, and computation time of the seven G × E set-based tests are investigated through simulations or real data analyses.

## Materials and Methods

### Adaptive Combination of Bayes Factors Method

Suppose a gene or an analysis region contains *L* SNPs. Let *Y* be the phenotype, *g*[·] be the link function, *G*_*l*_ be the number of minor allele (0, 1, or 2) at the *l*^th^ SNP (*l* = 1, …, *L*), *E* be the environmental factor, and ***X*** be the vector of potential confounder covariates. First, we assess each SNP × E interaction by considering the following generalized linear model (GLM):

(1) $$\begin{array}{l} g\left[E\left(Y\right)\right]=\beta_{0}+\beta_{G}G_{l}+\beta_{E}E+\beta_{GE}G_{l}E+{\beta^{\prime }}_{X}X,\;l=1,\cdots \;,L. \end{array}$$

For simplicity, we omit the subscript “*i*” that represents the data of the *i*^th^ subject. The SNP × E interaction is of interest, and therefore *H*_0_:β_*GE*_ = 0 vs. *H*_1_:β_*GE*_ ≠ 0. Let ${\hat{\beta }}_{GE}$ be the maximum likelihood estimate (MLE) of β_*GE*_. According to the asymptotic normality of MLE, ${\hat{\beta }}_{GE}$ follows a normal distribution with a mean of β_*GE*_ and a variance of *V*, i.e., ${\hat{\beta }}_{GE}\sim N\left(\beta_{GE},V\right)$.

We assume that the true interaction effects follow a normal distribution with a mean of 0 and a variance of *W*, i.e., β_*GE*_ ~ *N*(0, *W*). The Bayes factor (BF) (Wakefield, 2007, 2009) of the SNP × E interaction is

(2) $$\begin{array}{l} BF=\frac{\mathrm{Pr}\left(Data|H_{1}\right)}{\mathrm{Pr}\left(Data|H_{0}\right)}=\sqrt{\frac{\hat{V}}{\hat{V}+W}}\exp\left(\frac{{\hat{\beta }}_{GE}^{2}W}{2\hat{V}\left(\hat{V}+W\right)}\right), \end{array}$$

where ${\hat{\beta }}_{GE}^{\;}$ is the MLE of β_*GE*_, and $\hat{V}$ is the estimated variance of ${\hat{\beta }}_{GE}^{\;}$. To propose a prior that can be applicable to most situations, we first scale the environmental factor E to range from 0 to 1. A dichotomous E will be coded as 0 or 1 whereas a continuous E will be first scaled to be ${E_{\;}}^{\prime }=\left(E-E_{\min}\right)/\left(E_{\max}-E_{\min}\right)$, in which *E*_min_ and *E*_max_ are the minimum and maximum of E, respectively. In this way, *G*_*l*_*E* in Equation (1) will be between 0 and 2, in the same range as *G*_*l*_.

The Wellcome Trust Case Control Consortium GWAS (WTCCC, 2007) specified the prior for SNP main effects as β_*G*_ ~ *N*(0, *W*), where *W* = 0.2^2^ = 0.04. This prior implies that we believe 95% of odds ratios (ORs) range from exp(−2 × 0.2) = 0.67 to exp(2 × 0.2) = 1.49. Now that *G*_*l*_*E* is in the same range as *G*_*l*_, we consider using the same prior for SNP × E interaction, i.e., β_*GE*_ ~ *N*(0, *W*) where *W* = 0.2^2^ = 0.04. Reported SNP × E interactions have been of modest effect sizes that can be positive or negative (Simino et al., 2013; Rudolph et al., 2016; Sung et al., 2018), and therefore *N*(0, *W* = 0.04) may be a reasonable prior for β_*GE*_ (Lin et al., 2018).

To apply ADABF to continuous traits, we should first standardize the traits to have a mean of 0 and a standard deviation of 1, as implemented in our ADABF R code that can be downloaded from http://homepage.ntu.edu.tw/~linwy/ADABFGE.html. The prior of *N*(0, *W* = 0.04) implies that 95% of β_*G*_*E*__*l*__s range from (−2 × 0.2) = −0.4 to (2 × 0.2) = 0.4. This may also be a reasonable prior for β_*GE*_ when traits are continuous.

Because SNP × E interaction effects reported by empirical studies have been modest (Simino et al., 2013; Rudolph et al., 2016; Sung et al., 2018), this prior variance (*W* = 0.2^2^ = 0.04) may be slightly large for β_*G*_*E*__*l*__s. However, a larger prior variance can just reflect our uncertainty of the prior information (Wang et al., 2009).

After calculating the BFs of all the *L* SNP × E, we sort these *L* BFs from the largest to the smallest, and denote them as *BF*_(1)_ ≥ *BF*_(2)_ ≥ ⋯ ≥ *BF*_(*L*)_. The leading *k* BFs are summarized by $S_{k}=\sum_{l=1}^{k}\log\left(BF_{\left(l\right)}\right)$, where *k* = 1, ···, *L*. Let ${\hat{\beta }}_{GE,H_{0}}$ be the vector containing *L* ${\hat{\beta }}_{GE}^{\;}$s under the null hypothesis *H*_0_ (none of the *L* SNPs interact with E). We draw *B* sets of ${\hat{\beta }}_{GE,H_{0}}$ from the multivariate normal distribution with a mean vector of **0** and a variance-covariance matrix incorporating the pairwise linkage disequilibrium (LD) among the *L* SNPs, and then calculate $S_{k}^{\left(1\right)}$, …, $S_{k}^{\left(B\right)}$ accordingly. The details can be found from Lin et al. (2018).

By comparing *S*_*k*_ with its counterparts from *H*_0_ ($S_{k}^{\left(1\right)}$, …, $S_{k}^{\left(B\right)}$), we obtain the *P*-value regarding *S*_*k*_, where *k* = 1, ···, *L*. We then find the minimum *P*-values (across *k* = 1, ···, *L*) for the observed sample and for each of the resampling replicates. By comparing these minimum *P*-values, we obtain the significance of G × E for the observed sample. The efficient sequential resampling procedure (Liu et al., 2016) is used to speed up ADABF, in which the minimum and maximum numbers of resampling were set at 10^3^ and 10^7^, respectively. The resampling procedure is repeated until the *P* > 100/*B*, where *B* is the number of resampling.

Because the same prior variance *W* is used for the observed sample and for each of the resampling replicates, the performance of ADABF is robust to the selection of *W* (Lin et al., 2018). The R code of the ADABF method can be downloaded from http://homepage.ntu.edu.tw/~linwy/ADABFGE.html. A Perl script is also provided to facilitate genome-wide analyses, http://homepage.ntu.edu.tw/~linwy/ADABFGEfromPLINK.html.

### Set-Based Gene-EnviRonment InterAction Test (SBERIA)

There are two steps in SBERIA (Jiao et al., 2013): the filtering stage and the G × E stage. For case-control studies, a commonly-used filtering stage is to regress E on each SNP and assess the association of each SNP with E, by fitting a logistic regression for binary E or a linear regression for continuous E (Murcray et al., 2009; Jiao et al., 2013). This strategy is referred to as the “SNP-E association filtering.”

Suppose a positive interaction between SNP (coded as 0, 1, or 2) and E (coded as 0 or 1) is responsible for the susceptibility of a rare disease. Subjects with E = 1 and SNP = 2 will have an increased disease risk. If cases are ascertained, more E = 1 and SNP = 2 combinations will be observed in cases, representing that SNP and E will be positively associated in cases. Assuming SNP and E are approximately independent in controls, they will be also positively associated in the combined case-control data (Jiao et al., 2013). Similarly, if there is a negative interaction between SNP and E, they will be negatively associated in the combined case-control data. Therefore, for rare-disease studies with ascertained cases, the association between SNP and E in combined case-control samples can be an efficient filtering statistic for detecting SNP × E interaction (Murcray et al., 2011). Dai et al. (Proposition 3) has justified the validity of using this filtering stage in G × E studies (Dai et al., 2012).

In the subsequent G × E stage, the hypothesis of interest is *H*_0_:α_*GE*_ = 0 vs. *H*_1_:α_*GE*_ ≠ 0 in the following GLM,

(3) $$\begin{array}{l} g\left[E\left(Y\right)\right]=\alpha_{0}+{\alpha^{\prime }}_{G}G+\alpha_{E}E+\alpha_{GE}EG^{\prime }ŵ+{\alpha^{\prime }}_{X}X, \end{array}$$

where ***G*** is the vector of the numbers of minor allele (0, 1, or 2) at the *L* SNPs, and **ŵ** is the vector of weights given to the *L* SNPs. The weight is determined by the sign and significance of the filtering statistic. The weight given to a SNP is 1 if it is positively associated with E, −1 if it is negatively associated with E, and is a very small value (e.g., 0.0001) if the SNP is not statistically associated with E (i.e., filtering test *P*-value > a pre-specified significance level, say, 0.10 in Jiao et al., 2013). The SBERIA approach uses this weighting scheme because the SNP-E association test has been shown to be asymptotically independent of the SNP × E interaction test (Murcray et al., 2009; Dai et al., 2012) and is powerful for filtering (Jiao et al., 2013).

Another commonly-used screening strategy is the “main-effect filtering.” Each SNP is first screened by testing *H*_0_:γ_*G*_ = 0 vs. *H*_1_:γ_*G*_ ≠ 0 in the following GLM:

(4) $$\begin{array}{l} g\left[E\left(Y\right)\right]=\gamma_{0}+\gamma_{G}G_{l}+{\gamma^{\prime }}_{X}X. \end{array}$$

To preserve the type I error rates, the filtering statistic (stage 1) and the following interaction test statistic (stage 2) must be asymptotically independent under the null hypothesis. Dai et al. have proven the validity of using the “main-effect filtering” as the screening strategy (Dai et al., 2012). Each element in **ŵ** represents the weight given to a SNP, which is 1 if the SNP is positively associated with *Y*, −1 if it is negatively associated with *Y*, and is a very small value (0.0001) if the SNP is not statistically associated with *Y* (i.e., *P*-value > a pre-specified significance level, say, 0.10 in Jiao et al., 2013).

### Variance Component (VC) Test

The class of VC tests include iSKAT (Lin et al., 2016), GESAT (Lin et al., 2013), INT_FIX, INT_RAN, and JOINT (Chen et al., 2014). VC tests are based on the following GLM:

(5) $$\begin{array}{l} g\left[E\left(Y\right)\right]=\delta_{0}+{\delta^{\prime }}_{G}G+\delta_{E}E+{\delta^{\prime }}_{GE}S+{\delta^{\prime }}_{X}X, \end{array}$$

where $S={\left[\begin{array}{cccc} EG_{1} & EG_{2} & \cdots & EG_{L} \end{array}\right]}^{\prime }$. The vector $\delta_{GE}={\left[\begin{array}{cccc} \delta_{GE_{1}} & \delta_{GE_{2}} & \cdots & \delta_{GE_{L}} \end{array}\right]}^{\prime }$ contains the *L* SNP × E interaction effects. Assuming δ_*G*_*E*__*l*__s (*l* = 1, …, *L*) follow a distribution with a mean of 0 and a variance of τ_2_. The null hypothesis *H*_0_:**δ**_*GE*_ = 0 is then reduced to *H*_0_:τ_2_ = 0. The score statistic to test *H*_0_:τ_2_ = 0 vs. *H*_1_:τ_2_ > 0 can be referred to Equation (6) in Lin et al. (2016).

Similar among the five VC tests, δ_*G*_*E*__*l*__s (*l* = 1, …, *L*) are assumed to be random effects that follow a distribution. Therefore, testing *H*_0_:**δ**_*GE*_ = 0 can be reduced to testing *H*_0_:τ_2_ = 0. However, these five VC tests are dissimilar in two aspects.

First, they take different approaches to estimate the SNP main effects, δ_*G*_*l*__s (*l* = 1, …, *L*). INT_FIX treats δ_*G*_*l*__s as fixed effects, whereas INT_RAN assumes δ_*G*_*l*__s follow a distribution with a mean of 0 and a variance of τ_1_. GESAT and iSKAT use ridge regression to estimate δ_*G*_*l*__s under the null hypothesis of *H*_0_:**δ**_*GE*_ = 0. JOINT simultaneously tests whether SNP main effects or G × E interaction effects exist, i.e., *H*_0_:τ_1_ = τ_2_ = 0 vs. *H*_1_:τ_1_ > 0 or τ_2_ > 0. Therefore, it is not a pure test for detecting G × E.

Second, iSKAT allows an exchangeable correlation ρ among δ_*G*_*E*__*l*__s (*l* = 1, …, *L*) and searches for the optimal ρ. The other four VC tests all assume that δ_*G*_*E*__*l*__s are independent to each other (i.e., ρ = 0).

## Results

To reflect the real LD structures of the human genome, we used GWAS data from the TWB as our simulation material. The TWB aims to build a research database that integrates the genomic profiles, lifestyle patterns, dietary habits, and environmental exposures of residents aged 30–70 years in Taiwan (Chen et al., 2016). Community-based volunteers donated blood, took a physical examination, and completed a questionnaire with a face-to-face interview.

Most of these community-based volunteers were unrelated subjects. To exclude subjects with cryptic relatedness, we first estimated the genome-wide identity by descent (IBD) sharing coefficients among seemingly unrelated individuals from the whole-genome data. Using PLINK-1.9 (Purcell et al., 2007), we obtained the IBD scores for all pairs of subjects, i.e., PI-HAT = Pr(IBD = 2) + 0.5× Pr(IBD = 1). “PI-HAT” is a parameter used in PLINK to quantify pairwise IBD scores. Some GWAS excluded relatives within third-degree consanguinity, and therefore removed one person from a pair with PI-HAT ≥ 0.125 (Lowe et al., 2009; Mok et al., 2014). We here use a slightly more stringent threshold, 0.1. After removing subjects with cryptic relatedness (PI-HAT > 0.1), our analysis data included 16,555 unrelated subjects (8,213 males and 8,342 females).

The whole-genome genotyping of the TWB data revealed 631,941 autosomal SNPs. We excluded 22,212 SNPs with genotyping rates <95% and 5,988 SNPs with Hardy-Weinberg test *P* < 5.7 × 10^−7^ (WTCCC, 2007). The remaining 603,741 SNPs were used for the simulations and the following real data analysis. Because the SNP positions in the TWB data were based on the human genome GRCh37/hg19 assembly, we mapped the variants into genes according to the same assembly in the UCSC Genome Bioinformatics database (http://www.genome.ucsc.edu). In total, 24,769 autosomal genes were identified. Furthermore, following the conventional gene-based tests (Liu et al., 2010), we incorporated 50 kb in the 3′ and 5′ regions that might regulate a gene.

We assessed the type I error rates and power of the seven tests using simulations. Our ADABF was compared with rareGE (Chen et al., 2014), SBERIA (Jiao et al., 2013), GESAT (Lin et al., 2013), and iSKAT (Lin et al., 2016). These competitor methods have been developed with user-friendly analysis tools that are popular choices for G × E studies. The “rareGE” function in the “rareGE” R package (version 0.1) provides *P*-values for the following three tests: (1) INT_FIX: a G × E test that treats the SNP main effects as fixed effects; (2) INT_RAN: a G × E test that treats the SNP main effects as random effects; and (3) JOINT: a joint test of the genetic main effects and G × E interactions. Both GESAT (Lin et al., 2013) and iSKAT (Lin et al., 2016) were implemented using the “iSKAT” R package (version 1.2).

### Type I Error Rates

Given the genotypes of each subject from the TWB, his/her continuous trait was simulated according to

(6) $$\begin{array}{l} Y=\beta_{G}G_{l}+\beta_{GE}G_{l}E+e, \end{array}$$

where *G*_*l*_ is the minor allele count (0, 1, or 2) at the *l*^th^ SNP, *E* is the environmental factor, and *e* is the random error term following the standard normal distribution. Moreover, we simulated binary traits according to

(7) $$\begin{array}{l} \log\frac{\mathrm{Pr}\left(Y=1\right)}{1-\mathrm{Pr}\left(Y=1\right)}=-0.4+\beta_{G}G_{l}+\beta_{GE}G_{l}E, \end{array}$$

where the intercept was log (0.40.6)=−0.4, corresponding to a disease prevalence of 0.4, which was the worldwide prevalence of hypertension among adults aged ≥ 25 years (Abebe et al., 2015).

In Equations (6) and (7), E was a binary environmental factor taking a value of 0 or 1 each with a probability of 0.5. Because E was randomly sampled, the “SNP-E association filtering” (Murcray et al., 2009; Jiao et al., 2013) that computes the association between E and each SNP was inefficient. Therefore, in our simulations, we used the above-mentioned “main-effect filtering” (Dai et al., 2012) in SBERIA.

To evaluate the validity of these G × E tests, we let β_*G*_ = β_*GE*_ = 0 and generated the phenotypes of 16,555 subjects according to Equations (6) or (7). We repeated 41 rounds of genome-wide G × E analysis for the 24,769 autosomal genes so that each G × E test was evaluated at least one million times (24, 769 × 41 = 1, 015, 529). Following the conventional gene-based tests (Liu et al., 2010), we incorporated 50 kb in the 3′ and 5′ regions that might regulate a gene. The number of SNPs involved in a gene depends on the length of the gene. Table 1 presents the empirical type I error rates under various nominal significance levels based on 1,015,529 replications of the continuous traits and binary traits separately. All the tests preserved the type I error rates. Tables S1–S3 in our Supplementary Materials further present the type I error rates stratified by the number of SNPs involved in a gene. The results are similar to Table 1, indicating that the type I error rates do not much depend on the number of SNPs in a gene.

**Table 1:** Empirical type I error rates in the simulation study.

**Traits**	**Nominal significance levels**	**ADABF**	**INT_FIX**	**INT_RAN**	**JOINT**	**SBERIA**	**iSKAT**	**GESAT**
Continuous, β_*G*_ = β_*GE*_ = 0 assigned to Equation (6)	0.05	0.049807	0.050379	0.050163	0.050147	0.050389	0.052703	0.052583
	0.01	0.009549	0.009944	0.009882	0.010036	0.010081	0.011072	0.011570
	0.001	0.000927	0.000962	0.000948	0.001168	0.001008	0.001166	0.001300
	0.0001	0.000080	0.000088	0.000091	0.000156	0.000115	0.000125	0.000139
	5 × 10^−5^	0.000040	0.000039	0.000042	0.000068	0.000057	0.000061	0.000066
	2.5 × 10^−6^	0.000000	0.000001	0.000001	0.000002	0.000001	0.000002	0.000003
Binary, β_*G*_ = β_*GE*_ = 0 assigned to Equation (7)	0.05	0.050339	0.050739	0.050540	0.050385	0.049992	0.052701	0.053239
	0.01	0.008855	0.009989	0.009913	0.010193	0.010111	0.011149	0.011655
	0.001	0.000865	0.000972	0.000965	0.001200	0.001011	0.001191	0.001333
	0.0001	0.000082	0.000100	0.000105	0.000138	0.000103	0.000121	0.000146
	5 × 10^−5^	0.000054	0.000053	0.000058	0.000071	0.000054	0.000062	0.000076
	2.5 × 10^−6^	0.000003	0.000003	0.000003	0.000003	0.000003	0.000005	0.000004

*Each entry represents the proportion of P-values smaller than the corresponding nominal significance level based on 1,015,529 simulation replicates*.

We also evaluated the validity of these G × E tests in the presence of genetic main effects. If β_*GE*_ = 0 but β_*G*_ ≠ 0, all tests, except for JOINT, were valid (results not shown). Thus, if we obtain a significant test result using the JOINT method, we cannot know whether this significance is contributed by G × E or not. Therefore, the JOINT test should not be used if G × E is of the main interest. It is suitable for detecting genetic main effects while allowing for G × E.

### Power

The true number of SNPs interacting with E may not be large in the genome (McCarthy et al., 2008; Liu et al., 2016). Therefore, we simulated one or four non-null β_*GE*_s in a gene. To investigate the impact of the gene length on power, we randomly drew three genes (i.e., *CHD5, TNNT3*, and *RFX3*), respectively, incorporating 20, 50, and 100 SNPs, for simulations. Assuming *d* SNPs interact with E (*d* = 1 or 4), the continuous traits of the 16,555 subjects were generated according to

(8) $$\begin{array}{l} Y=\overset{d}{\underset{l=1}{\sum }}\beta_{G_{l}}G_{l}+\overset{d}{\underset{l=1}{\sum }}\beta_{GE_{l}}G_{l}E+e, \end{array}$$

where β_*G*_*l*__ is the SNP main effect, β_*G*_*E*__*l*__ is the effect size of SNP × E, *G*_*l*_ is the minor allele count (0, 1, or 2) at the *l*^th^ SNP that interacts with E (*l* = 1, …, *d*), and *e* is the random error term following the standard normal distribution. Moreover, the binary traits were simulated according to

(9) $$\begin{array}{l} \log\frac{\mathrm{Pr}\left(Y=1\right)}{1-\mathrm{Pr}\left(Y=1\right)}=-0.4+\overset{d}{\underset{l=1}{\sum }}\beta_{G_{l}}G_{l}+\overset{d}{\underset{l=1}{\sum }}\beta_{GE_{l}}G_{l}E. \end{array}$$

The magnitudes of SNP main effects (|β_*G*_*l*__|) and SNP × E interaction effects (|β_*G*_*E*__*l*__|) were evaluated at three levels: small, medium, and large. For continuous traits, the effect sizes were uniformly drawn from [0.08, 0.12] (small), [0.13, 0.17] (medium), and [0.18, 0.22] (large), respectively. For binary traits, the effect sizes were uniformly drawn from [log(1.05), log(1.15)] (small), [log(1.25), log(1.35)] (medium), and [log(1.45), log(1.55)] (large), respectively.

Table 2 lists the 11 simulation scenarios for power comparison, including 3 for *d* = 1 and 8 for *d* = 4. Scenarios (1-1), (4-1), and (4-5) are pure interaction models without SNP main effect. Scenarios (1-2), (4-2), and (4-6) include SNP × E interaction effects with SNP main effects in the same direction. Scenarios (1-3), (4-3), and (4-7) include SNP × E interaction effects with SNP main effects in the opposite direction. Scenarios (4-4) and (4-8) include SNP × E interaction effects with 50% SNP main effects in the same direction and 50% in the opposite direction.

**Table 2:** The 11 simulation scenarios for power comparison.

	**SNP main effects**				**SNP x E interaction effects**			
**Scenario**	**β_*G*__1_**	**β_*G*__2_**	**β_*G*__3_**	**β_*G*__4_**	**β_*G*__*E*__1_**	**β_*G*__*E*__2_**	**β_*G*__*E*__3_**	**β_*G*__*E*__4_**
(1-1)	0	0	0	0	+	0	0	0
	One positive SNP x E interaction effect without SNP main effect.							
(1-2)	+	0	0	0	+	0	0	0
	One positive SNP x E interaction effect, with SNP main effect in the same direction.							
(1-3)	−	0	0	0	+	0	0	0
	One positive SNP x E interaction effect, with SNP main effect in the opposite direction.							
(4-1)	0	0	0	0	+	+	+	+
	Four positive SNP x E interaction effects without SNP main effect.							
(4-2)	+	+	+	+	+	+	+	+
	Four positive SNP x E interaction effects, all with SNP main effects in the same direction.							
(4-3)	−	−	−	−	+	+	+	+
	Four positive SNP x E interaction effects, all with SNP main effects in the opposite direction.							
(4-4)	+	+	−	−	+	+	+	+
	Four positive SNP x E interaction effects, two with SNP main effects in the same direction and the other two in the opposite direction.							
(4-5)	0	0	0	0	+	+	−	−
	Two positive and two negative SNP x E interaction effects, without SNP main effect.							
(4-6)	+	+	−	−	+	+	−	−
	Two positive and two negative SNP x E interaction effects, all with SNP main effects in the same direction.							
(4-7)	−	−	+	+	+	+	−	−
	Two positive and two negative SNP x E interaction effects, all with SNP main effects in the opposite direction.							
(4-8)	+	−	+	−	+	+	−	−
	Two positive and two negative SNP x E interaction effects, two with SNP main effects in the same direction and the other two in the opposite direction.							

**Figure 1:**
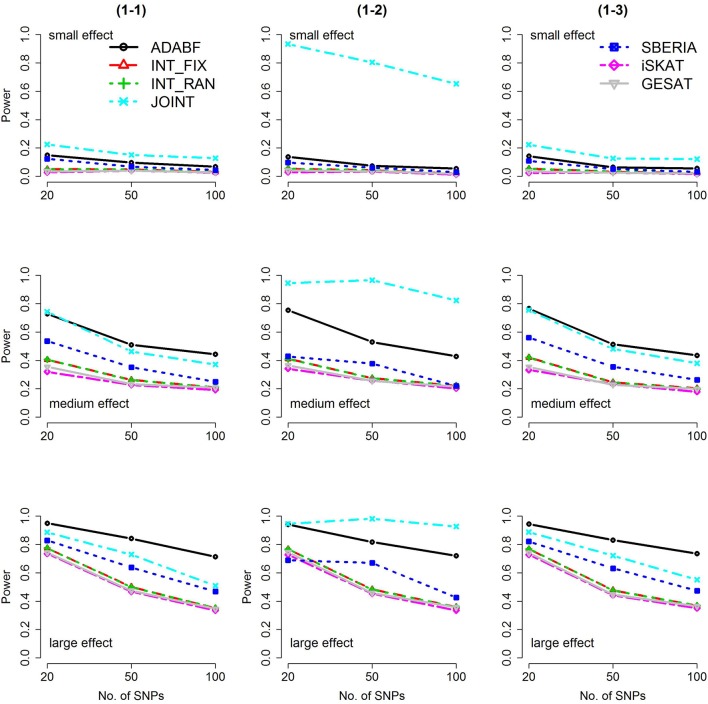
Power of the seven tests for continuous traits (1 SNP × E). The x-axis represents the number of SNPs in the gene, whereas the y-axis depicts the power at the nominal significance level α = 2.5 × 10^−6^. From the top row to the bottom row, the magnitudes of SNP main effects and SNP × E interaction effects were evaluated at three levels: small, medium, and large, respectively.

**Figure 2:**
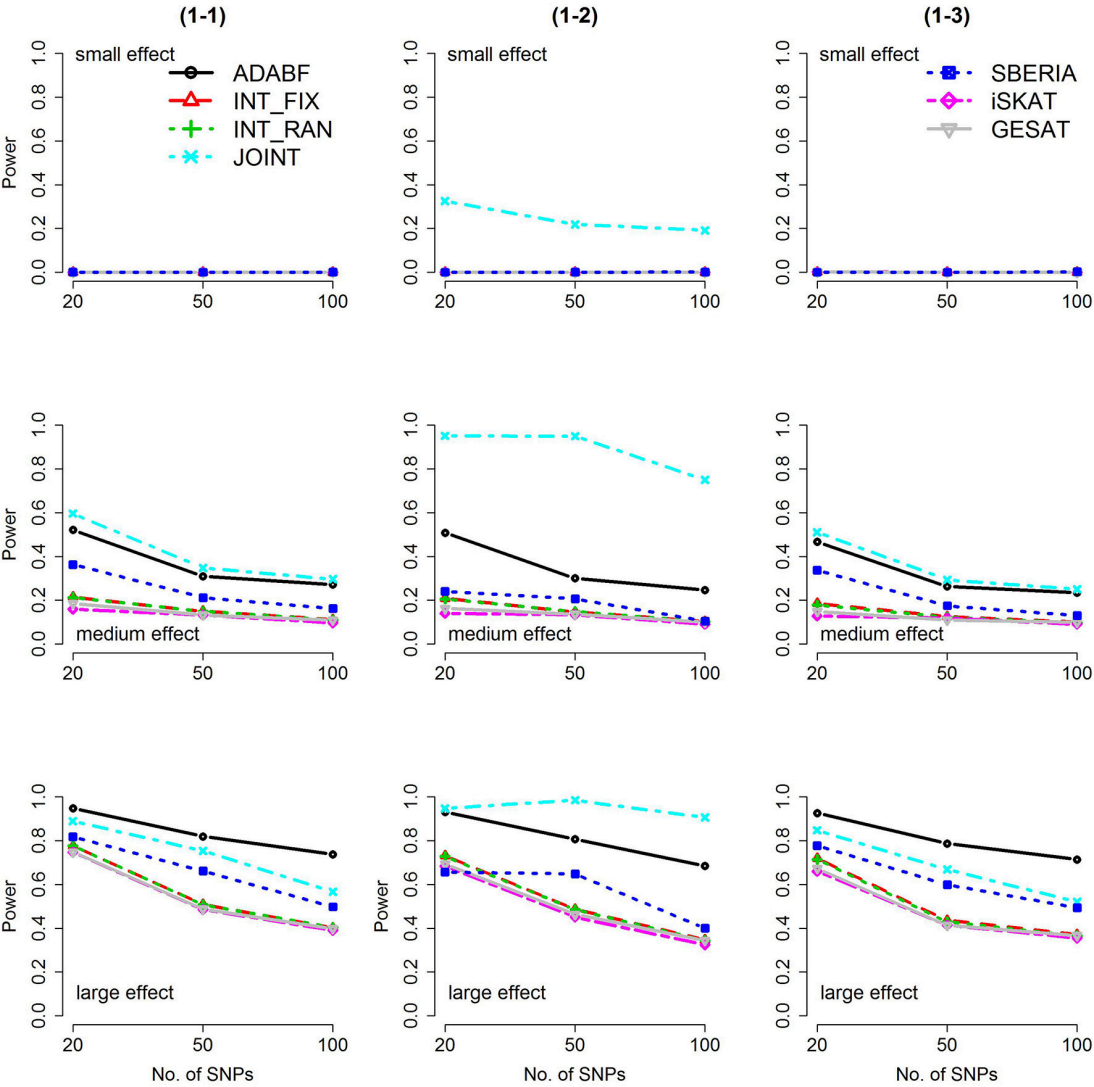
Power of the seven tests for binary traits (1 SNP × E). The x-axis represents the number of SNPs in the gene, whereas the y-axis depicts the power at the nominal significance level α = 2.5 × 10^−6^. From the top row to the bottom row, the magnitudes of SNP main effects and SNP × E interaction effects were evaluated at three levels: small, medium, and large, respectively.

**Figure 3:**
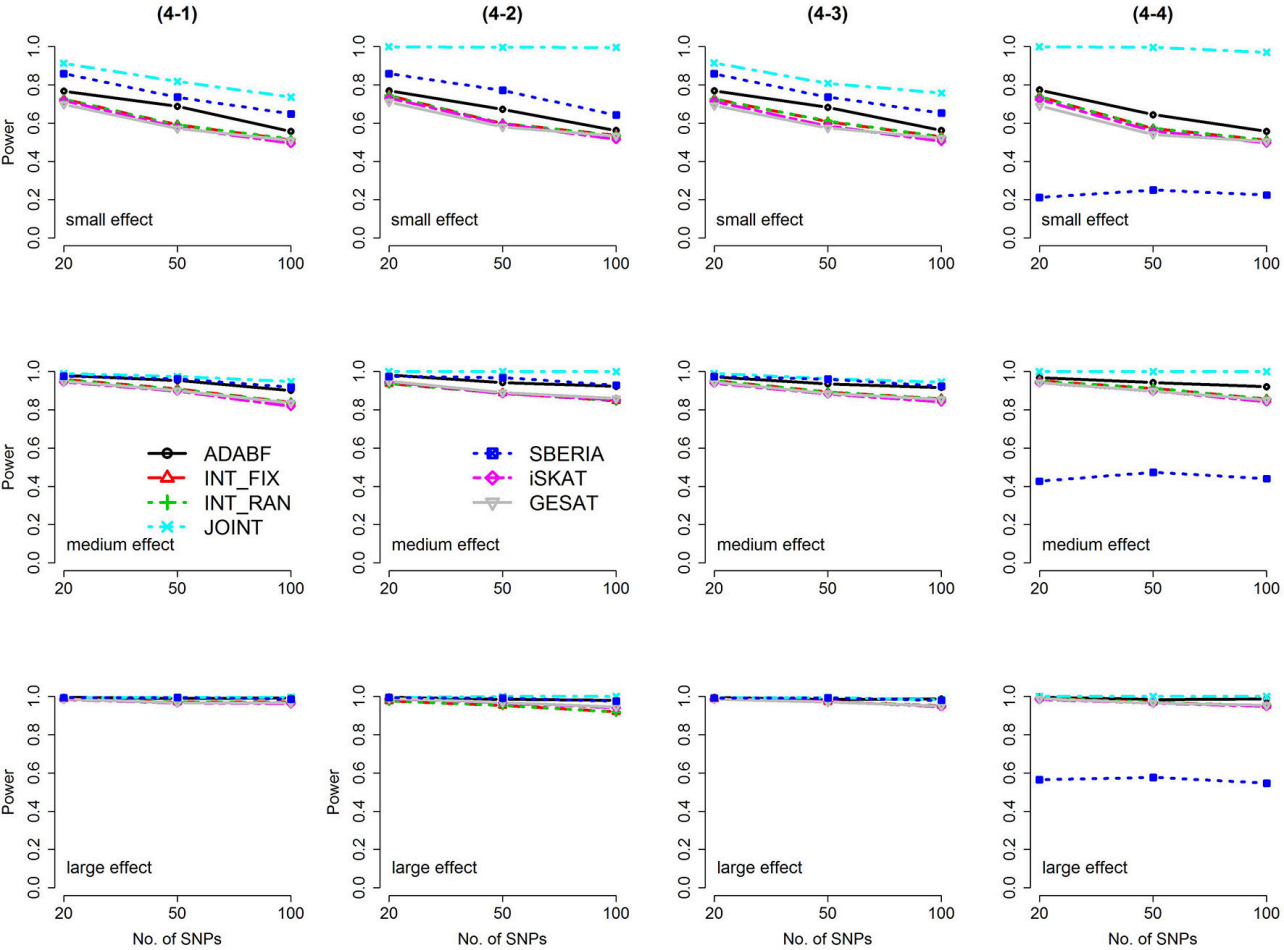
Power of the seven tests for continuous traits (4 SNP × E, from Scenario 4-1 to 4-4). The x-axis represents the number of SNPs in the gene, whereas the y-axis depicts the power at the nominal significance level α = 2.5 × 10^−6^. From the top row to the bottom row, the magnitudes of SNP main effects and SNP × E interaction effects were evaluated at three levels: small, medium, and large, respectively.

**Figure 4:**
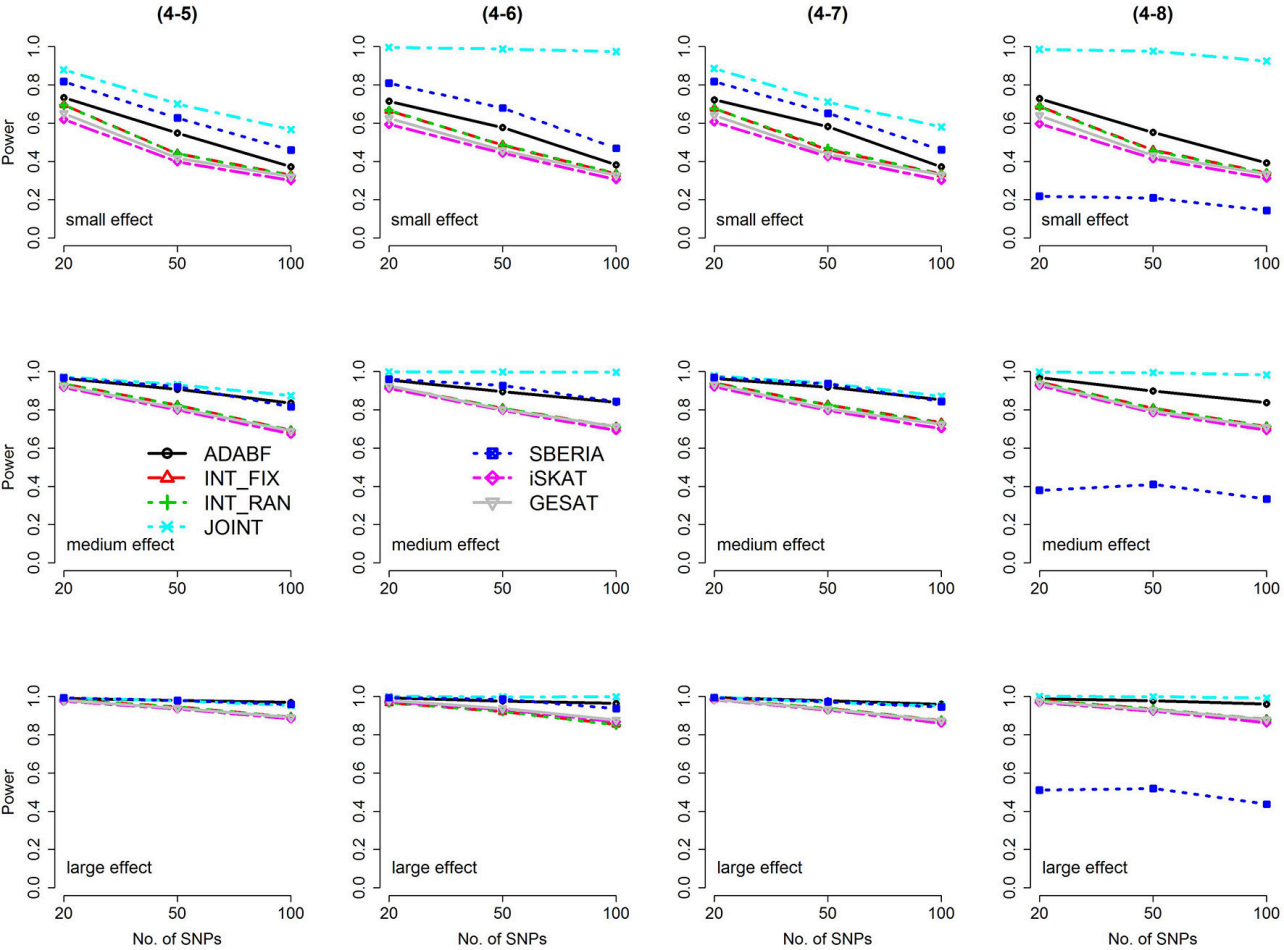
Power of the seven tests for continuous traits (4 SNP × E, from Scenario 4-5 to 4-8). The x-axis represents the number of SNPs in the gene, whereas the y-axis depicts the power at the nominal significance level α = 2.5 × 10^−6^. From the top row to the bottom row, the magnitudes of SNP main effects and SNP × E interaction effects were evaluated at three levels: small, medium, and large, respectively.

**Figure 5:**
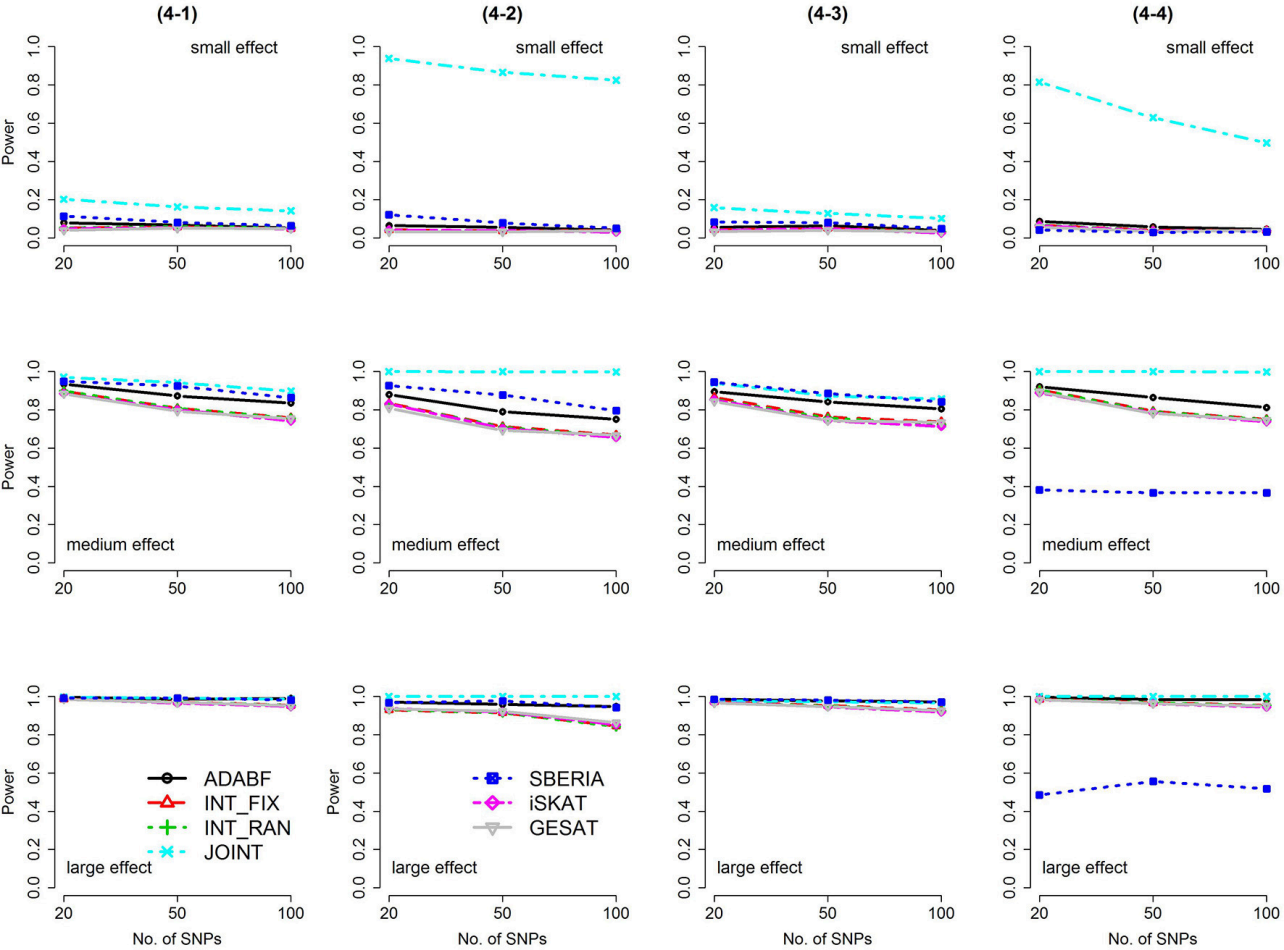
Power of the seven tests for binary traits (4 SNP × E, from Scenario 4-1 to 4-4). The x-axis represents the number of SNPs in the gene, whereas the y-axis depicts the power at the nominal significance level α = 2.5 × 10^−6^. From the top row to the bottom row, the magnitudes of SNP main effects and SNP × E interaction effects were evaluated at three levels: small, medium, and large, respectively.

**Figure 6:**
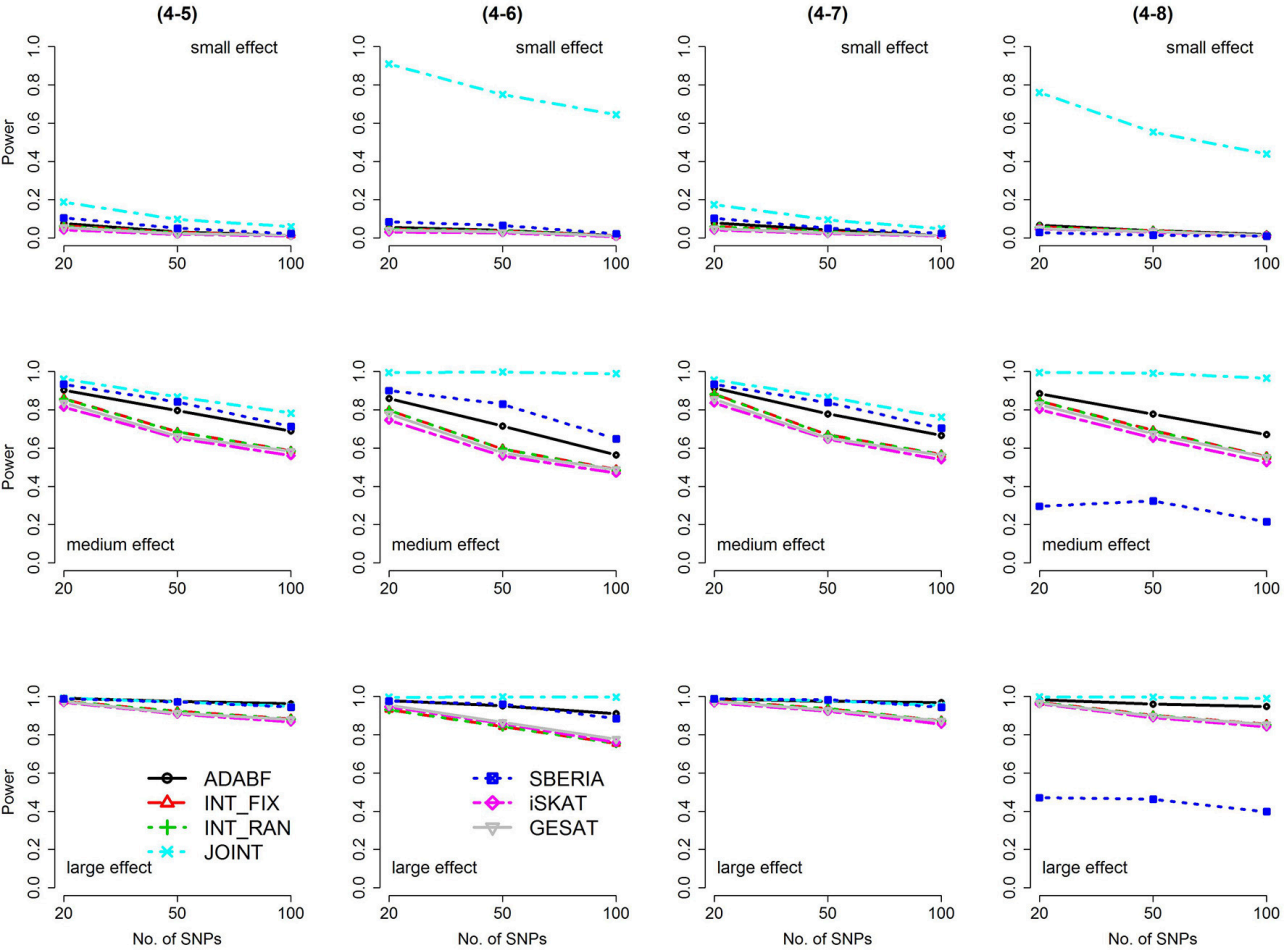
Power of the seven tests for binary traits (4 SNP × E, from Scenario 4-5 to 4-8). The x-axis represents the number of SNPs in the gene, whereas the y-axis depicts the power at the nominal significance level α = 2.5 × 10^−6^. From the top row to the bottom row, the magnitudes of SNP main effects and SNP × E interaction effects were evaluated at three levels: small, medium, and large, respectively.

Based on 1,000 replications for each scenario, Figures 1, 2 present the results of 1 SNP × E (i.e., *d* = 1) for continuous and binary traits, respectively. The results of 4 SNP × E (i.e., *d* = 4) are shown in Figures 3, 4 (for continuous traits) and Figures 5, 6 (for binary traits). Under the same scenario and the same level of effect sizes, the power of all tests decreased as the number of SNPs increased. This was because the proportion of non-null β_*GE*_s was decreasing as the number of SNPs increased. For example, when *d* = 4, the proportions of non-null β_*GE*_s were 4/20, 4/50, and 4/100, respectively.

The JOINT test was generally the most powerful test. However, as mentioned above, it is not a pure G × E test. Among the 6 pure G × E tests, ADABF was more powerful under 1 SNP × E (i.e., *d* = 1, Figures 1, 2). Let *m* be the number of SNPs in a gene, where *m* = 20, 50, or 100 in our power comparison. When *d* = 1, *m* − 1 SNPs exhibit no interactions with E. ADABF outperformed the other tests because it excluded SNP × E with smaller BF; thus, ADABF was more robust to the inclusion of many (*m* − 1) null β_*GE*_s.

Among the 6 pure G × E tests, SBERIA can be more powerful than ADABF under 4 SNP × E (i.e., *d* = 4, Figures 3–6). However, SBERIA suffered from a power loss in Scenarios (4-4) and (4-8), where 50% SNP main effects were in the same direction with the SNP × E interaction effects while 50% were in the opposite direction. This is because SBERIA builds a G × E term by incorporating the SNPs that pass the filtering stage (i.e., *E*G′**ŵ** in Equation 3). The weight (elements in **ŵ**) given to a SNP is 1 if it is positively associated with *Y*, −1 if it is negatively associated with *Y*, and is a very small value (e.g., 0.0001) if the SNP is not statistically associated with *Y*. When 50% SNP main effects were in the same direction with the SNP × E interaction effects while 50% were in the opposite direction, the positive and negative SNP × E interactions in *E*G′**ŵ** were canceled out. Therefore, SBERIA suffered from a power loss in Scenarios (4-4) and (4-8).

### Application to the Taiwan Biobank Data

Subsequently, we applied these G × E methods to the TWB data. Among the TWB subjects, ~79.9% were of the southern Han Chinese ancestry, ~5% were of the northern Han Chinese ancestry, and ~14.5% belonged to a third group (Chen et al., 2016). To adjust for the population substructure, the 603,741 SNPs that passed the quality-control stage were used to construct the principal components (PCs). We aim to explore the interaction effects between genes and alcohol consumption on blood pressure levels. Our study was approved by the Research Ethics Committee of National Taiwan University Hospital (NTUH-REC no. 201612188RINA).

In the TWB data, “alcohol drinking” is defined as a weekly alcohol intake >150 c.c. for at least 6 months. Among the 16,555 subjects, 14,779 subjects answered “no” to alcohol drinking, whereas 1,764 subjects answered “yes.” Totally 12 subjects did not respond to this question. Therefore, the environmental factor (“alcohol drinking”) was binary here. Both DBP and SBP were measured twice in a sitting position, with a 5-min interval between the two measurements. As suggested by Jamieson et al. (1990) and others (Husemoen et al., 2008), two measurements of blood pressure should routinely be taken, and the average recorded. Therefore, in the following analysis, we used the average of the two measurements of DBP (or SBP) as the phenotype.

Prior to the G × E analysis, we first regressed DBP (the average of two measured DBPs) and SBP (the average of two measured SBPs) on gender, age, alcohol drinking, body mass index (BMI), and the first seven PCs. In Table 3, we list the regression coefficients regarding gender, age, alcohol drinking, and BMI. Males, elder subjects, subjects consuming alcohol, and subjects with larger BMI exhibit a significantly higher mean blood pressure than females, younger subjects, subjects without alcohol consumption, and subjects with smaller BMI. On average, alcohol drinking results in an increase of ~1.51 mmHg in DBP and ~2.10 mmHg in SBP. This finding that an increased alcohol intake elevates blood pressures is consistent with the conclusions of numerous studies (Xin et al., 2001; Puddey and Beilin, 2006; Tomson and Lip, 2006).

**Table 3:** The regression models for the DBP and SBP analyses (prior to the G × E analysis).

	**DBP****^a^**				**SBP****^a^**			
**Important explanatory variables in the regression model**	**Regression coefficient** ($\hat{\beta }$)	**Standard error of** $\hat{\beta }$	**Wald statistic**, ${\;}^{\hat{\beta }}\text{​​}╱\text{​}{\text{​}}_{s.e.\left(\hat{\beta }\right)}\;$	***P*****-value**	**Regression coefficient** ($\hat{\beta }$)	**Standard error of** $\hat{\beta }$	**Wald statistic**, ${\;}^{\hat{\beta }}\text{​​}╱\text{​}{\text{​}}_{s.e.\left(\hat{\beta }\right)}\;$	***P*****-value**
Gender^b^ (1: female; 0: male)	−5.8385	0.1613	−36.188	< 2 × 10^−16^	−5.7753	0.2440	−23.669	< 2 × 10^−16^
Age^c^(in year, continuous variable)	0.1380	0.0069	19.961	< 2 × 10^−16^	0.6018	0.0105	57.579	< 2 × 10^−16^
Alcohol drinking^d^ (1: yes; 0: no)	1.5107	0.2552	5.920	3.29 × 10^−9^	2.0961	0.3860	5.431	5.69 × 10^−8^
Body mass index (BMI)^e^(in kg/m^2^, continuous variable)	0.8884	0.0215	41.380	< 2 × 10^−16^	1.2633	0.0325	38.907	< 2 × 10^−16^

a *The first seven PCs were also adjusted in the model*.

b *Interpretation of gender, Males have significantly higher mean blood pressure than females*.

c *Interpretation of age, Elder subjects have significantly higher mean blood pressure than younger subjects*.

d *Interpretation of alcohol drinking, Subjects consuming alcohol have significantly higher mean blood pressure than subjects without alcohol consumption*.

e *Interpretation of BMI, Subjects with larger BMI have significantly higher mean blood pressure than subjects with smaller BMI*.

#### Single Marker Analysis

The first strategy to detect G × E is single SNP analysis. Let *Y* be DBP or SBP, *G*_*l*_ be the number of minor allele (0, 1, or 2) at the *l*^th^ SNP, *E* be the environmental factor (“alcohol drinking”), and ***X*** be the vector of covariates, including age, gender, BMI, and the first seven PCs. We fitted a linear regression for each of the 603,741 SNPs,

(10) $$\begin{array}{l} E\left(Y\right)=\beta_{0}+\beta_{G}G_{l}+\beta_{E}E+\beta_{GE}G_{l}E+{\beta^{\prime }}_{X}X,l=1,\cdots \;,603741. \end{array}$$

This single marker analysis was performed using PLINK (version 1.9) (Purcell et al., 2007). PLINK reported low genomic inflation factors after adjusting the first seven PCs, i.e., λ_*GC*_ = 1.081 for DBP and 1.058 for SBP. As suggested by GWAS such as Li et al. (2015), these λ_*GC*_s represented minimal effects of population stratification. We then tested *H*_0_:β_*GE*_ = 0 vs. *H*_1_:β_*GE*_ ≠ 0, and compared the *P*-value with the commonly-used genome-wide significance level, 5 × 10^−8^. No significant SNP × E were identified for DBP or SBP.

#### Gene-Based Analysis

Then, we performed the seven gene-based tests. According to the human genome GRCh37/hg19 assembly, there are 24,769 autosomal genes. We followed the conventional gene-based tests (Liu et al., 2010), and therefore incorporated 50 kb in the 3′ and 5′ regions that might regulate genes. The “main-effect filtering” and “SNP-E association filtering” were both used in the SBERIA approach, and they were referred to as “SBERIA1” and “SBERIA2,” respectively.

1. SBERIA1 (main-effect filtering): In the filtering stage, a linear regression was fitted for each SNP, i.e., $E\left(Y\right)=\gamma_{0}+\gamma_{G}G_{l}+{\gamma^{\prime }}_{X}X$. The validity of using this filtering stage was justified by Corollary 1 proposed by Dai et al. (2012). Using this filtering strategy into Jiao et al.'s SBERIA approach, when the *P*-value of testing *H*_0_:γ_*G*_ = 0 vs. *H*_1_:γ_*G*_ ≠ 0 was smaller than 0.1, the weight given to the *l*^th^ SNP was 1 if ${\hat{\gamma }}_{G}>0$ and was −1 if ${\hat{\gamma }}_{G}<0$ (${\hat{\gamma }}_{G}$ was the MLE of γ_*G*_). When the *P*-value of testing *H*_0_:γ_*G*_ = 0 vs. *H*_1_:γ_*G*_ ≠ 0 was larger than 0.1, the weight given to the *l*^th^ SNP was 0.0001 (Jiao et al., 2013).
2. SBERIA2 (SNP-E association filtering): In the filtering stage, a logistic regression was fitted for each SNP, i.e., $\log it\left(E\right)=\delta_{0}+\delta_{G}G_{l}+{\delta^{\prime }}_{X}X$, where *E* = “alcohol drinking” was binary. The validity of using this filtering stage was justified by Proposition 3 of Dai et al. (2012). According to Jiao et al.'s SBERIA approach, when the *P*-value of testing *H*_0_:δ_*G*_ = 0 vs. *H*_1_:δ_*G*_ ≠ 0 was smaller than 0.1, the weight given to the *l*^th^ SNP was 1 if ${\hat{\delta }}_{G}>0$ and was −1 if ${\hat{\delta }}_{G}<0$ (${\hat{\delta }}_{G}$ was the MLE of δ_*G*_). When the *P*-value of testing *H*_0_:δ_*G*_ = 0 vs. *H*_1_:δ_*G*_ ≠ 0 was larger than 0.1, the weight given to the *l*^th^ SNP was 0.0001 (Jiao et al., 2013).

Table 4 lists the genes that are significant according to at least one of the analysis methods, where the statistical significance is claimed if a *P* < 2.5 × 10^−6^, where $2.5\times 10^{-6}{=}^{0.05}{/}_{20000}$ is the commonly-used genome-wide significance level in gene-based analyses (Epstein et al., 2015). Regarding DBP, the *ADAMTS7P1* gene was identified by the GESAT test (*P* = 9.5 × 10^−7^). At this gene, the *P*-values provided by other 6 tests all reached the suggestive significance level (*P*<$5\times 10^{-5}{=}^{1}{/}_{20000}$). The other genes listed in Table 4 were presumably to have genetic main effects rather than G × E interactions, because they were only identified by the JOINT test.

**Table 4:** Significant genes (*P*-value < 2.5 × 10^−6^) identified by at least one of the G × E tests.

**Phenotype**	**Gene**	**Chr**.	**Analysis region**^***a***^	**#(SNPs)**	***P*****-values (highlighted if smaller than the genome-wide significance level** **=** **2.5 × 10**^**−6**^**)**								***GxE*** ^***d***^
					**ADABF**	**INT_FIX**	**INT_RAN**	**JOINT**	**SBERIA1**^***b***^	**SBERIA2**^***c***^	**iSKAT**	**GESAT**	
DBP	*CCDC66*	3	56541184–56705864	14	0.94	0.71	0.72	3.9 × 10^−7^	0.42	0.34	0.55	0.74	
	*PRDM8*	4	81055033–81175483	49	5.7 × 10^−4^	7.6 × 10^−4^	6.1 × 10^−4^	1.4 × 10^−6^	1.4 × 10^−2^	0.42	1.1 × 10^−3^	5.2 × 10^−4^	
	*FGF5*	4	81137742–81262171	41	0.02	0.12	0.11	7.1 × 10^−10^	0.71	1.00	0.20	0.11	
	*ADAMTS7P1*	15	82535621–82676915	4	6.5 × 10^−6^	6.0 × 10^−6^	5.7 × 10^−6^	6.5 × 10^−6^	2.4 × 10^−5^	1.9 × 10^−5^	3.7 × 10^−6^	9.5 × 10^−7^	Yes
	*BICDL2*	16	3027683–3136950	8	4.4 × 10^−3^	6.0 × 10^−3^	6.0 × 10^−3^	1.5 × 10^−6^	0.02	0.03	5.6 × 10^−3^	2.4 × 10^−3^	
	Time spent (in hours) ^e^				82		470		8	10	330	312	
SBP	*FGF5*	4	81137742–81262171	41	0.12	0.17	0.17	3.2 × 10^−11^	0.73	0.69	0.29	0.17	
	*ATP2B1*	12	89931826–90153130	27	0.59	0.58	0.56	2.1 × 10^−7^	0.78	0.38	0.49	0.61	
	*ATP2B1-AS1*	12	90052732–90155729	12	0.68	0.59	0.57	8.8 × 10^−8^	0.96	0.16	0.22	0.62	
	Time spent (in hours) ^e^				78		463		8	10	327	310	

a *The analysis regions were based on the human GRCh37/hg19 assembly. Following the conventional gene-based tests, we also incorporated 50 kb in the 3′ and 5′ regions that might regulate the gene*.

b SBERIA1, SBERIA coupled with “main-effect filtering.”

c SBERIA2, SBERIA coupled with “SNP-E association filtering.”

d *Only ADAMTS7P1 was supported to have interaction effects with alcohol consumption, on DBP. The other genes listed in this table were suggested to have genetic main effects because they were only identified by the JOINT test*.

e *The total time for analyzing all the 24,769 autosomal genes*.

**Figure 7:**
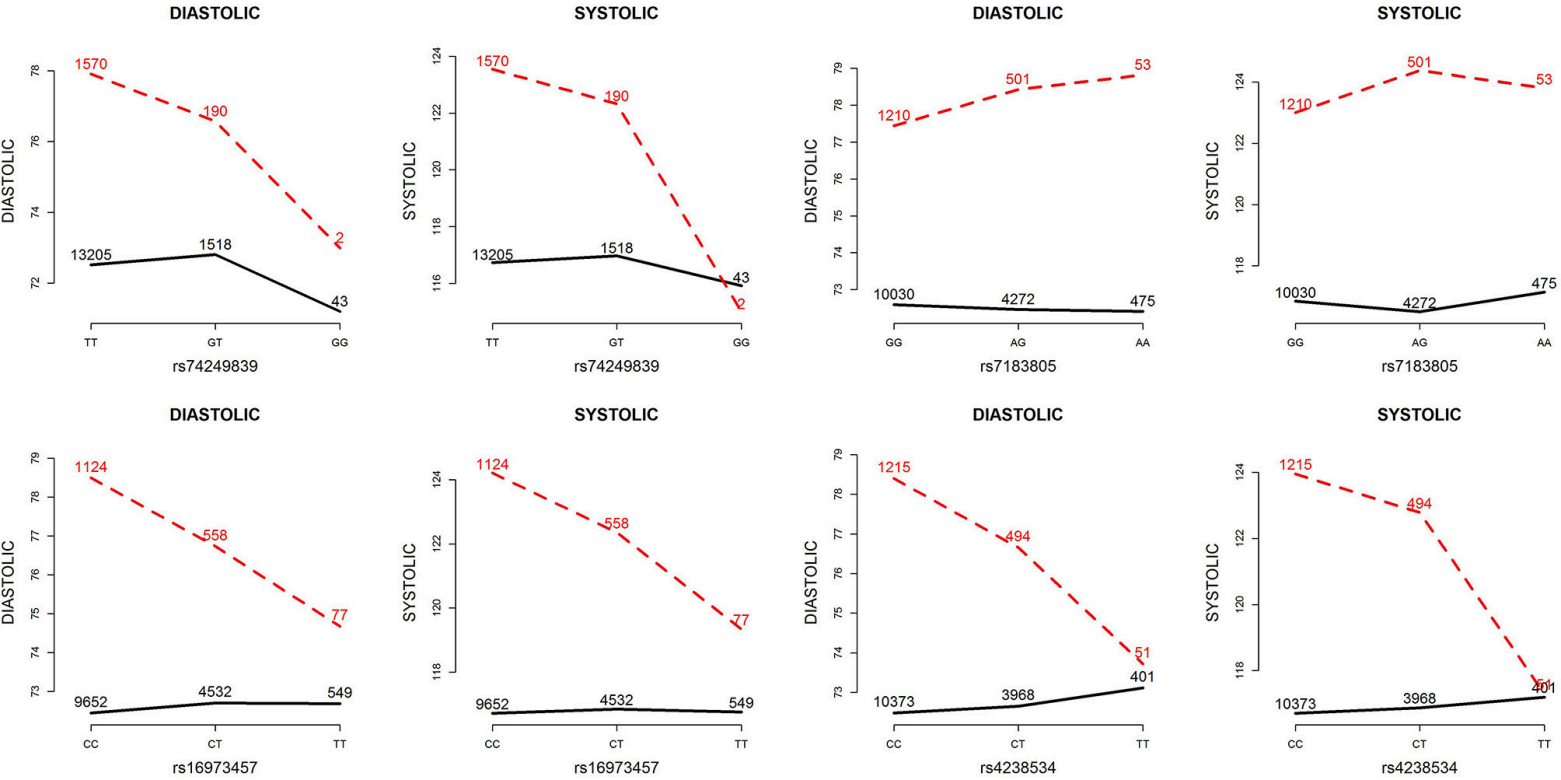
Plots of SNP×alcohol interaction effects on DBP and SBP. These are the interaction plots of the four SNPs in Table 5. As shown in these plots, the SNP×alcohol interaction patterns in DBP are similar to those in SBP. The black curves depict the mean of DBP or SBP among the non-drinkers, whereas the red dashed curves depict that among the drinkers. The number shown on each point represents the sample size of that category.

Table 5 presents the information regarding the four SNPs in the analysis region of the *ADAMTS7P1* gene. For DBP analysis, two BFs of SNP× alcohol interactions were >100 (representing decisive evidence against the null hypothesis Jeffreys, 1961; Kass and Raftery, 1995), including rs16973457 and rs4238534. Plots of the SNP × alcohol interaction effects on DBP and SBP are presented in Figure 7. Here, non-drinkers (black curves) exhibit similar blood pressure values across different genotypes. However, drinkers (red dashed curves) exhibit elevated blood pressure if they possess certain genotypes. Interestingly, if we ignore *G*_*l*_*E* from Equation (10), the main effects of these SNPs are not significant (shown in the final column of Table 5). This finding highlights the importance of considering the SNP × alcohol interaction effect on blood pressure.

**Table 5:** The four SNPs in the analysis region of the *ADAMTS7P1* gene.

**Phenotype**	**SNP**	**Position (base pair)**	**Minor allele**	**Major allele**	**MAF**	**SNP** **×** **alcohol interaction test**^**a**^					**If SNP × alcohol interaction was not incorporated in the model**^**b**^
						**${\hat{\beta }}_{GE}$**	**$s.e.\left({\hat{\beta }}_{GE}\right)$**	**Wald statistic**	***P*-value (*H*_0_:β_*GE*_ = 0 vs. *H*_1_:β_*GE*_ ≠ 0**)	**Bayes factor**	***P*-value (*H*_0_:β_*G*_ = 0 vs. *H*_1_:β_*G*_ ≠ 0**)
DBP	rs74249839	82537997	G	T	0.0544	−1.5477	0.7757	−1.995	0.0460	1.95	0.58
	rs7183805	82539431	A	G	0.1761	0.8042	0.4595	1.750	0.0801	0.88	0.82
	rs16973457	82563991	T	C	0.1924	−1.8607	0.4309	−4.318	1.59 × 10^−5^	1519.09	0.65
	rs4238534	82564555	T	C	0.1627	−2.1048	0.4642	−4.535	5.81 × 10^−6^	3883.43	0.76
SBP	rs74249839	82537997	G	T	0.0544	−1.6558	1.1731	−1.411	0.1581	0.78	0.99
	rs7183805	82539431	A	G	0.1761	0.9474	0.6949	1.363	0.1728	0.48	0.76
	rs16973457	82563991	T	C	0.1924	−2.4675	0.6518	−3.786	0.000154	186.85	0.19
	rs4238534	82564555	T	C	0.1627	−2.4668	0.7024	−3.512	0.000446	74.07	0.56

a *The DBP (or SBP) was regressed by Equation (10) and β_GE_ was of the main interest*.

b *If we ignored G_l_E from Equation (10), the main effects of these four SNPs were not significant*.

#### Computation Time

As shown in Table 4, we also provide the time spent for analyzing the 24,769 autosomal genes, using a Linux platform with a Dell Intel Xeon E5-2690 2.9 GHz processor and 8 GB memory. SBERIA (8~10 h for a phenotype) is the fastest method, followed by ADABF (~80 h). iSKAT and GESAT both required more than 300 h. INT_FIX, INT_RAN, and JOINT were conducted using a function in the “rareGE” package, and these three tests totally required more than 400 h.

## Discussion

Environmental factors, such as diet, exercise, alcohol intake and tobacco use, can modify the associations of genetic variants with disease (Lee et al., 2011). G × E can shed light on biological processes leading to disease, identify high-risk subjects, and improve disease prediction (Hunter, 2005; Dudbridge and Fletcher, 2014).

Our ADABF method has been proposed as a powerful polygenic approach to detect G × E (Lin et al., 2018). This method can also serve as a gene-based G × E test. In this study, we compare our ADABF method with six existing gene-based tests, through extensive simulations and real data analyses. Our ADABF method is among the most powerful tests. Although the JOINT test is typically the most powerful method, it is not appropriate for assessing G × E in the presence of genetic main effects. As presented by Table 4, although seven genes were identified as significant by JOINT, none of them were replicated by any of the six pure G × E tests at the genome-wide significance level ($2.5\times 10^{-6}{=}^{0.05}{/}_{20000}$) or at the suggestive significance level ($5\times 10^{-5}{=}^{1}{/}_{20000}$). The JOINT test should not be used if G × E is of the main interest, but it is useful in detecting genetic main effects while allowing for G × E.

Notably, all gene-based tests can be performed using a pre-specified weighting scheme for SNPs. For example, if rare variants are believed to have stronger interactions with E, the beta distribution density function with parameters 1 and 25 evaluated at the sample MAF, i.e., *Beta*(*MAF*; 1, 25), is commonly used to weight the SNPs (Wu et al., 2011; Lin et al., 2016). However, to present a fair comparison, we do not impose any additional weighting on these seven tests.

The *ADAMTS7P1* gene at 15q25.2 has not been reported to be associated with blood pressures or hypertension. Indeed, after removing the SNP×alcohol interaction (*G*_*l*_*E*) from Equation (10), the main effects of all the four SNPs within *ADAMTS7P1* were not significant. Although the *ADAMTS7P1*-alcohol interaction effect on SBP did not achieve the suggestive significance level ($5\times 10^{-5}{=}^{1}{/}_{20000}$), the *P*-values of the 6 pure G × E tests were all <10^−3^ (Table 6). Moreover, as presented in the bottom part of Table 5, the BF of rs16973457× alcohol interaction on SBP was >100 (representing decisive evidence against the null hypothesis Jeffreys, 1961; Kass and Raftery, 1995). Further gene×alcohol studies investigating this chromosome region will be warranted.

**Table 6:** Analysis of the *ADAMTS7P1*-alcohol interaction effect on SBP.

**Gene**	**Chr**.	**Analysis region**	**#(SNPs)**	***P*****-values**							
				**ADABF**	**INT_FIX**	**INT_RAN**	**JOINT**	**SBERIA1**	**SBERIA2**	**iSKAT**	**GESAT**
*ADAMTS7P1*	15	82535621–82676915	4	3.5 × 10^−4^	2.2 × 10^−4^	2.0 × 10^−4^	1.3 × 10^−3^	5.0 × 10^−4^	1.7 × 10^−4^	2.0 × 10^−4^	8.2 × 10^−5^

In this study, we extend our ADABF to G × E detection and compare it with six existing tests. Their validity, power, robustness, and computation time are investigated. SBERIA builds a G × E term by incorporating the SNPs that pass the filtering stage (i.e., *E**G*****′ŵ** in Equation 3); ADABF removes the SNP × E with smaller BFs. Both approaches take the advantage of screening out noises, and therefore they are usually more powerful than other pure G × E tests (Figures 1–6). However, it is worth noting that SBERIA suffers from a power loss when 50% SNP main effects are in the same direction with the SNP × E interaction effects while 50% are in the opposite direction. Considering the validity, power performance, robustness, and computation time, ADABF is recommended for genome-wide G × E analyses.

To detect G × E on a genome-wide scale, ADABF polygenic test (Lin et al., 2018) and ADABF gene-based test are two strategies with different aims. The ADABF polygenic test combines all SNPs that pass the pruning and filtering stages into a test, and therefore it does not suffer from a power loss due to the multiple-testing correction. A *P* < 0.05 or 0.01 is sufficient to reject *H*_0_ of no polygenic G × E interactions (Pan et al., 2015). By contrast, the power of ADABF gene-based test is compromised by the penalty of multiple testing. A *P*<$2.5\times 10^{-6}{=}^{0.05}{/}_{20000}$ is required to claim a significant gene-based test (Epstein et al., 2015). Despite a much more stringent significance threshold, the ADABF gene-based test can make statistical inference for specific chromosomal regions, whereas the ADABF polygenic test (Lin et al., 2018) make an inference for SNPs (passing the pruning and filtering stages) spread out the whole genome.

## Author Contributions

W-YL developed the ADABF method and the analysis tool, designed and performed the simulation study, analyzed the TWB data, and wrote the manuscript. C-CH contributed to the review and coding for the competitor methods. Y-LL, S-JT, and P-HK contributed to the writing of the manuscript, and provided the TWB data. All authors reviewed the manuscript.

### Conflict of Interest Statement

The authors declare that the research was conducted in the absence of any commercial or financial relationships that could be construed as a potential conflict of interest.
