# Supplementary material for: Genome-Wide Gene-Environment Interaction Analysis Using Set-Based Association Tests
Source: Front Genet. 2019 Jan 14;9:715. doi: 10.3389/fgene.2018.00715 (PMC6339974; doi:10.3389/fgene.2018.00715)
Supplement: Supplementary file 1 [file Data_Sheet_1.PDF]

Genome-wide gene-environment interaction analysis using set-based association tests (Supplementary Material)

Wan-Yu Lin [1,2]*, Ching-Chieh Huang [1], Yu-Li Liu [3], Shih-Jen Tsai [4,5], Po-Hsiu Kuo [1,2]

[1] Institute of Epidemiology and Preventive Medicine, College of Public Health, National Taiwan University, Taipei, Taiwan
[2] Department of Public Health, College of Public Health, National Taiwan University, Taipei, Taiwan
[3] Center for Neuropsychiatric Research, National Health Research Institutes, Zhunan, Miaoli County, Taiwan
[4] Department of Psychiatry, Taipei Veterans General Hospital, Taipei, Taiwan
[5] Division of Psychiatry, National Yang-Ming University, Taipei, Taiwan

Traits	Nominal significance levels	ADABF	INT_FIX	INT_RAN	JOINT	SBERIA	iSKAT	GESAT
Continuous	**0.05**	0.04983	0.04979	0.04979	0.04949	0.05031	0.05234	0.05242
	**0.01**	0.00983	0.00969	0.00972	0.01003	0.01006	0.01131	0.01134
	**0.001**	0.00101	0.00092	0.00091	0.00114	0.00108	0.00124	0.00130
	**0.0001**	0.00011	0.00010	0.00010	0.00015	0.00012	0.00015	0.00016
Binary	**0.05**	0.05089	0.05023	0.05014	0.04987	0.04970	0.05291	0.05264
	**0.01**	0.00896	0.00974	0.00972	0.01020	0.01020	0.01126	0.01153
	**0.001**	0.00090	0.00090	0.00089	0.00116	0.00087	0.00119	0.00126
	**0.0001**	0.00009	0.00011	0.00011	0.00015	0.00009	0.00013	0.00015

Table S1. Empirical type I error rates in the simulation study (number of SNPs in a gene <= 20)

Each entry represents the proportion of *P*-values smaller than the corresponding nominal significance level based on 489,609 simulation replicates.

Traits	Nominal significance levels	ADABF	INT_FIX	INT_RAN	JOINT	SBERIA	iSKAT	GESAT
Continuous	**0.05**	0.05002	0.05098	0.05067	0.05066	0.05063	0.05306	0.05300
	**0.01**	0.00935	0.01011	0.01001	0.00995	0.01007	0.01088	0.01185
	**0.001**	0.00084	0.00098	0.00096	0.00120	0.00093	0.00105	0.00131
	**0.0001**	0.00006	0.00008	0.00008	0.00016	0.00012	0.00010	0.00012
Binary	**0.05**	0.04983	0.05096	0.05065	0.05047	0.04983	0.05236	0.05352
	**0.01**	0.00891	0.01021	0.01017	0.01027	0.01003	0.01117	0.01183
	**0.001**	0.00088	0.00106	0.00107	0.00125	0.00108	0.00119	0.00146
	**0.0001**	0.00008	0.00009	0.00010	0.00013	0.00012	0.00011	0.00014

Table S2. Empirical type I error rates in the simulation study (20 < number of SNPs in a gene <= 60)

Each entry represents the proportion of *P*-values smaller than the corresponding nominal significance level based on 424,009 simulation replicates.

Traits	Nominal significance levels	ADABF	INT_FIX	INT_RAN	JOINT	SBERIA	iSKAT	GESAT
Continuous	0.05	0.04879	0.05071	0.04986	0.05116	0.04977	0.05298	0.05163
	0.01	0.00905	0.01050	0.01012	0.01042	0.01022	0.01071	0.01150
	0.001	0.00088	0.00109	0.00109	0.00117	0.00100	0.00128	0.00126
	0.0001	0.00004	0.00007	0.00007	0.00019	0.00009	0.00013	0.00011
Binary	0.05	0.04980	0.05225	0.05199	0.05249	0.05208	0.05313	0.05492
	0.01	0.00814	0.01024	0.00979	0.00983	0.00998	0.01053	0.01151
	0.001	0.00063	0.00092	0.00090	0.00116	0.00140	0.00118	0.00114
	0.0001	0.00007	0.00009	0.00009	0.00011	0.00012	0.00011	0.00015

Table S3. Empirical type I error rates in the simulation study (number of SNPs in a gene > 60)

Each entry represents the proportion of *P*-values smaller than the corresponding nominal significance level based on 101,911 simulation replicates.
